# Supplementary material for: Community based reference interval of selected clinical chemistry parameters among apparently healthy Adolescents in Mekelle City, Tigrai, Northern Ethiopia
Source: PLoS One. 2020 Apr 7;15(4):e0231017. doi: 10.1371/journal.pone.0231017 (PMC7138298; doi:10.1371/journal.pone.0231017)
Supplement: Attachment — Submitted filename: Reviewer(Daniel Asmelash) Comments.docx [file pone.0231017.s001.docx]

Daniel Asmelash (Questions/Comments/Suggestions)

1. The title the authors worked with is interesting and sensible for African region. However, Thorough proof reading needed
2. Justify the research gap of this study, particularly why you focus on this specific age group?
3. Representativeness of the study? Does study done only on Mekelle city will infer for the whole Tigray region? Are you inferring for the general population of Tigray region?
4. What new thing your study added from the study by Molla Abebe et. al _(_<https://journals.plos.org/plosone/article?id=10.1371/journal.pone.0201782>)
5. Socio-demographic Characteristics of the study participants lacks depth, it needs more information
6. On the Data Collection and Laboratory analysis section of the manuscript you didn’t include laboratory methods analysis for each analyte?
7. On the data analysis why, you choose Mann Whitney U test non– parametric analysis?
8. I didn’t see what type of laboratory tests was done for your exclusion criteria and the number of study participants excluded by your exclusion criteria mainly Based on laboratory findings?
9. Abreviations should be clearly mentioned on the tables
10. The Discussion needs more clarification on the possible reasons of the findings.
